# AAV-Mediated Expression of miR-17 Enhances Neurite and Axon Regeneration In Vitro

**DOI:** 10.3390/ijms25169057

**Published:** 2024-08-21

**Authors:** Raquel Alves Almeida, Carolina Gomes Ferreira, Victor Ulysses Souza Matos, Julia Meireles Nogueira, Marina Pimenta Braga, Lucas Caldi Gomes, Erika Cristina Jorge, Frederico Marianetti Soriani, Uwe Michel, Vinicius Toledo Ribas

**Affiliations:** 1 Department of Morphology, Institute of Biological Sciences, Federal University of Minas Gerais, Av. Pres. Antônio Carlos, 6627, Belo Horizonte 31279-901, Brazilecjorge@icb.ufmg.br (E.C.J.); 2 Department of Genetics, Ecology and Evolution, Institute of Biological Sciences, Federal University of Minas Gerais, Belo Horizonte 31279-901, Brazilfredsori@icb.ufmg.br (F.M.S.); 3 Clinical Department of Neurology, TUM School of Medicine, Rechts der Isar Hospital, Technical University of Munich, 81675 Munich, Germany; lucas.caldi-gomes@tum.de; 4 Department of Neurology, University Medicine Göttingen, 37075 Göttingen, Germany

**Keywords:** axon, damage, regeneration, miR-17

## Abstract

Neurodegenerative disorders, including traumatic injuries to the central nervous system (CNS) and neurodegenerative diseases, are characterized by early axonal damage, which does not regenerate in the adult mammalian CNS, leading to permanent neurological deficits. One of the primary causes of the loss of regenerative ability is thought to be a developmental decline in neurons’ intrinsic capability for axon growth. Different molecules are involved in the developmental loss of the ability for axon regeneration, including many transcription factors. However, the function of microRNAs (miRNAs), which are also modulators of gene expression, in axon re-growth is still unclear. Among the various miRNAs recently identified with roles in the CNS, miR-17, which is highly expressed during early development, emerges as a promising target to promote axon regeneration. Here, we used adeno-associated viral (AAV) vectors to overexpress miR-17 (AAV.miR-17) in primary cortical neurons and evaluate its effects on neurite and axon regeneration in vitro. Although AAV.miR-17 had no significant effect on neurite outgrowth and arborization, it significantly enhances neurite regeneration after scratch lesion and axon regeneration after axotomy of neurons cultured in microfluidic chambers. Target prediction and functional annotation analyses suggest that miR-17 regulates gene expression associated with autophagy and cell metabolism. Our findings suggest that miR-17 promotes regenerative response and thus could mitigate neurodegenerative effects.

## 1. Introduction

Neurodegeneration in the central nervous system (CNS), as occurs in traumatic injuries, stroke, and neurodegenerative diseases, is a pathophysiological process characterized by the early degeneration of axons that frequently precedes somatic cell death [1]. Moreover, the regenerative ability of adult mammalian CNS neurons following an injury is severely restricted. In contrast, neurons from the immature CNS are able to regenerate their axons, an ability that is lost during development [2]. The failure of regenerative ability is assumed to be mainly caused by a developmental decline in neurons’ intrinsic capacity for axon growth, in addition to the formation of an inhibitory CNS environment [3]. The developmental loss in the capacity for axon regeneration has been shown in different regions of the CNS, including the Purkinje cells of the cerebellum [4], neurons from the brainstem [5], and the retinal ganglion cells [2]. Several molecules, including PTEN (phosphatase and tensin homolog), SOCS3 (suppressor of cytokine signaling 3), ROCK2 (Rho-associated coiled-coil-containing protein kinase 2) and many transcription factors such as KLF4, KLF9, SOX11, c-Myc, STAT3, c-Jun, and ATF3 have been identified as important regulators of axonal regeneration [6]. Specifically, members of the KLF family of transcription factors have been suggested to play roles in the developmental decline of the ability for axon re-growth [7].

Despite abundant evidence that transcription factors can regulate axon growth and regeneration in the CNS, the role of microRNAs (miRNAs), which are also modulators of gene expression, in these events remains less explored. MiRNAs are short, single-stranded, non-coding RNAs, ranging from 19 to 25 nucleotides in length, that play crucial roles in numerous cellular processes [8]. MiRNAs are involved in post-transcriptional regulation of gene expression by targeting messenger RNAs (mRNAs) by binding via their seed sequences to the 3′UTR or even coding regions [8]. A large number of studies have demonstrated altered miRNA expression levels in various neurodegenerative disorders [9], suggesting that these molecules may play crucial roles in such conditions. Among the various miRNAs recently identified with roles in the CNS is miR-17, emerging as an important regulator of biological processes and signaling pathways.

MiR-17 belongs to the miR-17-92 cluster, which includes five other mature miRNAs: miR-18a, miR-19a, miR-19b, miR-20a, and miR-92a [10]. In the cerebral cortex of mice, miR-17 is highly expressed during early development, and its expression levels gradually decrease as the cortex develops, especially in the postnatal period [11]. Moreover, overexpression of miR-17-5p promotes neuronal differentiation of neural progenitor cells through the downregulation of bone morphogenetic protein 2 (BMP2) signaling pathway [11], which is known to inhibit neurite outgrowth [12]. The decreased expression of miR-17 during the postnatal period, a time when neurons lose their intrinsic axonal regeneration capacity, and the negative regulation of the BMP2 signaling pathway by miR-17 suggest that miR-17 may play a role in controlling axonal outgrowth. However, the role of miR-17 in axonal outgrowth and, especially, axon regeneration remains unclear.

To further understand the role of miR-17 in neurite outgrowth and regeneration, we used adeno-associated virus (AAV) vectors to overexpress miR-17 in primary cortical neurons. We found that miR-17 overexpression did not affect neurite outgrowth and arborization. Nevertheless, AAV-mediated miR-17 overexpression enhances neurite and axon regeneration. Target prediction analysis suggested that miR-17 regulates the expression of genes involved in autophagy and cell metabolism. These data suggest that miR-17 might be an important target to promote a regenerative response, potentially constituting a promising therapeutic strategy for neurodegenerative disorders.

## 2. Results

### 2.1. AAV.miR-17 Efficiently Transduce Primary Cortical Neurons

**Figure 1:**
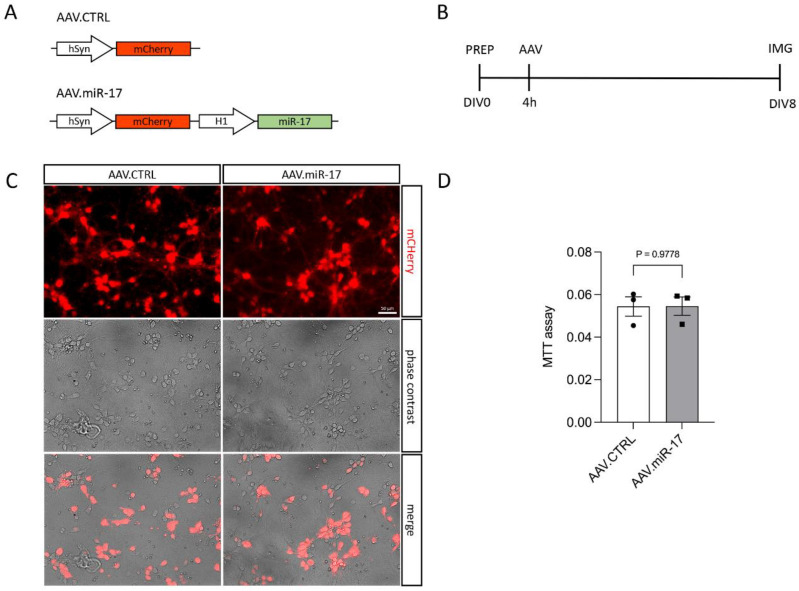
Transduction of primary cortical neurons with AAV.miR-17 and AAV.CTRL. (**A**) Vector maps of the control AAV (AAV. CTRL) and the AAV to overexpress the miR-17 (AAV.miR-17), both of them express the mCherry fluorophore under the control of a human synapsin (hSyn) promoter. The miR-17 expression is under the control of the H1 promoter. (**B**) Scheme of experimental setup for testing the transduction of AAV vectors. PREP: day of preparation of E18 rat cortical neurons; DIV: day in vitro; AAV: transduction with AAV vectors; IMG: imaging. (**C**) Representative images of cortical neurons 8 days after transduction with AAV.CTRL or AAV.miR-17 vectors showing the expression of the mCherry fluorescent protein (red), phase contrast, and the merge. Scale bar: 50 µm. (**D**) Quantification of cell viability through the MTT assay at the DIV8. (n = 3 independent cultures). Data are presented as single data points and means ± SEM. *p* = 0.9778, according to a two-tailed unpaired *t*-test.

To overexpress miR-17, we generated an AAV vector expressing the mCherry fluorescent protein under the control of the hSyn promoter and miR-17 under the control of the H1 promoter (AAV.miR-17). As a control, we generated an AAV vector, which expresses only the mCherry fluorescent protein (AAV.CTRL) (Figure 1A). Both vectors were used to transduce rat primary cortical neurons (Figure 1B–D). First, we confirmed that the AAV.miR-17 and AAV.CTRL were both able to transduce rat primary cortical neurons with around 90% transduction efficiency, as evaluated by the expression of mCherry by fluorescent microscopy 8 days after AAV transduction (Figure 1C). We used the expression of mCherry as a proxy for miR-17 expression. To assess potential AAV vector toxicity, we performed the MTT assay to evaluate cell viability. No significant differences in toxicity were detectable between AAV.miR-17 and AAV.CTRL (Figure 1D). In summary, we found that both AAV vectors efficiently transduce rat primary cortical neurons with no differential toxicity.

### 2.2. AAV.miR-17 Does Not Affect Neurite Outgrowth and Arborization

**Figure 2:**
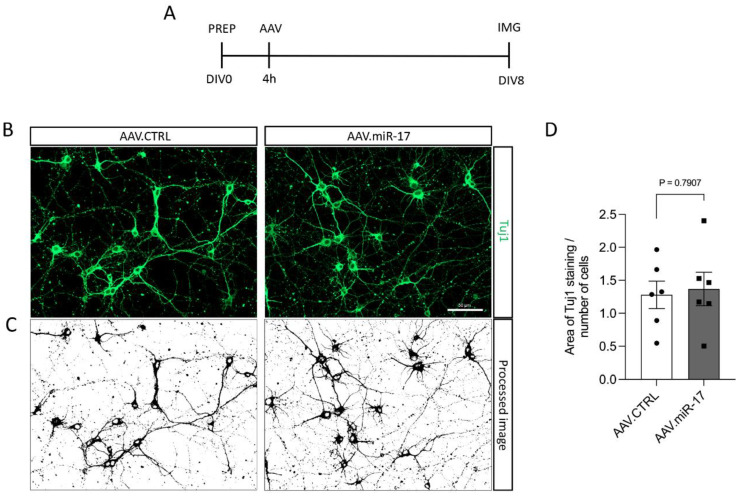
AAV.miR-17 has no effect on neurite outgrowth. (**A**) Scheme of experimental setup for neurite outgrowth assay. PREP: day of preparation of E18 rat cortical neurons; DIV: day in vitro; AAV: transduction with AAV vectors; IMG: imaging. (**B**) Representative images of DIV8 cortical neurons transduced with AAV.miR-17 or AAV.CTRL vectors showing the staining for β-III-tubulin (Tuj1, green). Scale bar: 50 μm. (**C**) The same representative images are shown in (**B**) after image processing for the quantification of neurite outgrowth. (**D**) Quantification of the area of the Tuj1 staining normalized by the number of cell bodies. (n = 6 independent cultures). Data are presented as single data points and means ± SEM. *p* = 0.7907, according to a two-tailed unpaired *t*-test.

To assess whether the overexpression of miR-17 had an influence on neurite outgrowth, we transduced rat primary cortical neurons with either AAV.miR-17 or AAV.CTRL and 8 days later, we fixed the cells and stained the neuronal cell bodies and neurites with an antibody against β-III-tubulin (Tuj1) (Figure 2A,B). Then, we imaged the cortical neurons in a fluorescent microscope, quantified the area occupied by the Tuj1 staining, and normalized by the number of neurons in each image. No significant differences were detectable in the area of the Tuj1 staining after transduction with AAV.miR-17 (1.4 ± 0.25) compared with AAV.CTRL (1.3 ± 0.21) (Figure 2C,D).

**Figure 3:**
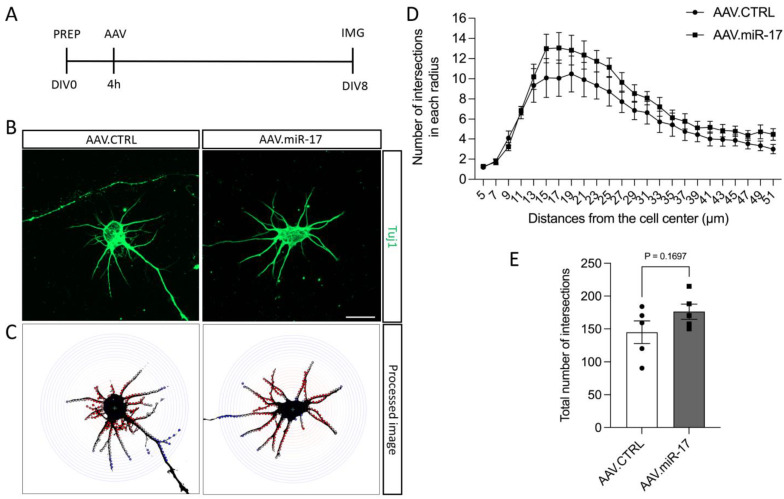
The complexity of the neurite arborization is not influenced by AAV.miR-17. (**A**) Scheme of the experimental setup for neurite arborization complexity assay. PREP: day of preparation of E18 rat cortical neurons; DIV: day in vitro; AAV: transduction with AAV vectors; IMG: imaging. (**B**) Representative images of DIV8 isolated cortical neurons transduced with AAV.miR-17 or AAV.CTRL vectors showing the staining for β-III-tubulin (Tuj1, green). Scale bar: 20 μm. (**C**) The same representative images are shown in (**B**) after image processing for the quantification of Sholl analysis, showing the concentric circles and the intersection between them and the neurites. (**D**) Quantification of the number of intersections between the neurites and the concentric circles at the given radius. Data are presented as means ± SEM (n = 5 independent cultures). (**E**) Quantification of the total number of intersections between the neurites and all the concentric circles. (n = 5 independent cultures). Data are presented as single data points and means ± SEM. *p* = 0.1697, according to a two-tailed unpaired *t*-test.

We also investigated whether AAV.miR-17 might have an influence on neurite arborization. To assess that, we used the same paradigm described before, but we seeded the neurons in a low density to be able to have isolated neurons, and we used the Sholl analysis to quantify neurite arborization complexity (Figure 3A–C). Quantification of the number of neurite intersections that occur at different distances from the soma in concentric circles showed that there were no significant differences between AAV.miR-17 and AAV.CTRL at any distance analyzed (Figure 3D). The total number of intersections among all distances analyzed was also not significantly different after transduction with AAV.miR-17 (176 ± 12) compared with AAV.CTRL (145 ± 17) (Figure 3E). Together, these results show that overexpression of miR-17 had no influence on neurite outgrowth and arborization.

### 2.3. AAV.miR-17 Enhances Neurite and Axon Regeneration

**Figure 4:**
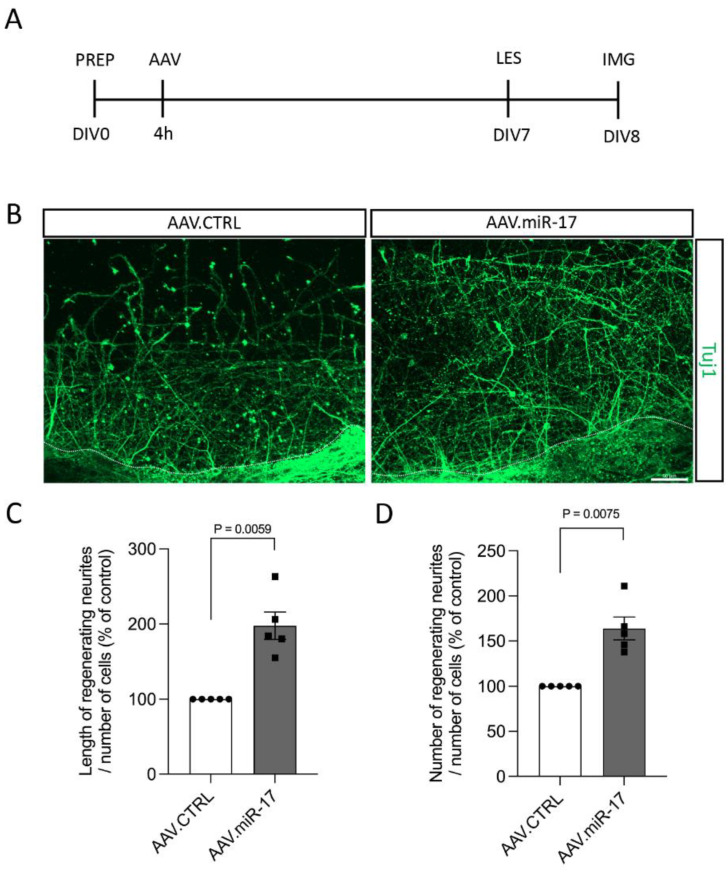
AAV.miR-17 enhances neurite regeneration after scratch lesions. (**A**) Scheme of the experimental setup for neurite regeneration assay. PREP: day of preparation of E18 rat cortical neurons; DIV: day in vitro; AAV: transduction with AAV vectors; LES: scratch lesion; IMG: imaging. (**B**) Representative images of cortical neurons transduced with AAV.CTRL or AAV.miR-17 vector and 24 h after the scratch lesion showing the regenerating neurites stained for β-III-tubulin (Tuj1, green). The dashed line indicates the scratch lesion border. Scale bar: 50 μm. (**C**) Quantification of the neurite length in the lesion area in a distance from 100 µm to 200 µm from the scratch border, normalized by the control group (AAV.CTRL). (**D**) Quantification of the number of neurites that re-growth until the distance of 200 µm from the scratch border, normalized by the control group (AAV.CTRL). (n = 5 independent cultures). Data are presented as single data points and means ± SEM. Indicated *p*-value according to one-sample *t*-test.

Although we did not find any difference in neurite outgrowth and arborization after miR-17 overexpression in non-lesioned neurons, lesions to the CNS can trigger different injury response signaling [13], and thus miR-17 could have an influence on neurite and axon re-growth. For the assessment of the pro-regenerative potential of miR-17, first, we used a scratch lesion model to evaluate neurite regeneration. Primary cortical neurons were transduced with either AAV.miR-17 or AAV.CTRL. Seven days later, a mechanical scratch lesion was performed, and 24 h later, the neurons were fixed and stained for beta-III-tubulin (Tuj1) (Figure 4A). The neurite length and numbers were quantified in the lesioned area and normalized by the number of cell bodies from the lesion border to 100 µm before the lesion. We found that transduction with AAV.miR-17 significantly enhances the length of regenerating neurites in the lesion area, with an increase of 198% (±18) compared to AAV.CTRL (Figure 4B,C). We also found a significant increase of 164% (±13) in the number of regenerating neurites at a 200 µm distance from the scratch lesion border compared to AAV.CTRL (Figure 4B,D).

**Figure 5:**
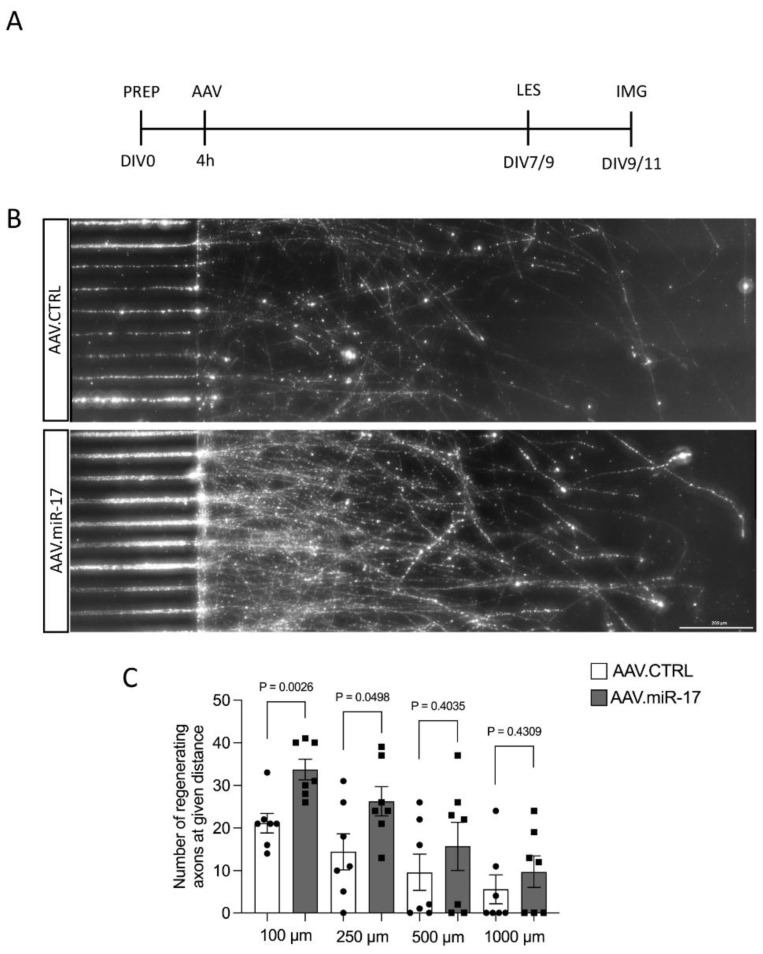
AAV.miR-17 increases axonal regeneration after axotomy. (**A**) Scheme of experimental setup for axonal regeneration analysis in microfluidic chambers. PREP: day of preparation of E18 rat cortical neurons; DIV: day in vitro; AAV: transduction with AAV vectors; LES: axotomy; IMG: imaging. (**B**) Representative images of cortical neurons transduced with AAV.CTRL or AAV.miR-17 vector and seeded in microfluidic chambers 48 h after the axotomy, showing the regenerating axons labeled with the mCherry fluorescent protein (white). Scale bar: 200 μm. (**C**) Quantification of the number of axons that re-growth until the given distances from the microgroove exit. (n = 7 independent cultures). Data are presented as single data points and means ± SEM. Indicated *p*-value according to two-tailed unpaired *t*-test.

Having found increased neurite regeneration by AAV.miR-17, we next aimed to examine whether overexpression of miR-17 might also promote the regeneration specifically of axons. We assessed the regeneration specifically of axons because, in the previous paradigm, we cannot exclude that the regenerating neurites are dendrites instead of axons and also because the most important process to regenerate after a lesion is the axon. To assess that, we used a previously established paradigm of selective axonal lesions of cortical neurons cultured in a microfluidic device [14,15]. We seeded rat primary cortical neurons in microfluidic devices and transduced them with either AAV.miR-17 or AAV.CTRL. After 7 to 9 days in culture, we selectively performed a lesion to the axons and studied axonal re-growth via live imaging at 48 h (Figure 5A). Axons were identified by the expression of mCherry. We counted the number of regenerating axons at defined distances (100–1000 μm) from the distal aperture of the microgrooves. We observed the re-growth of axons after lesion for both AAV vectors (Figure 5B). Neurons transduced with AAV.miR-17 showed a significant enhancement in the number of regenerating axons at 100 μm (34 ± 2.4) and 250 μm distance (26 ± 3.4) compared to AAV.CTRL (100 μm: 21 ± 2.3; 250 μm: 14 ± 4.2) (Figure 5C). There was no significant difference between AAV.miR-17 and AAV.CTRL in the distance of 500 µm (AAV.CTRL: 10 ± 4.3 vs. AAV.miR-17: 16 ± 5.6) and 1000 µm (AAV.CTRL: 6 ± 3.4 vs. AAV.miR-17: 10 ± 3.7). In summary, these data showed that AAV.miR-17 leads to an increased regenerative response of neurites and axons in vitro.

### 2.4. miR-17 Is Predicted to Regulate the Expression of Genes Involved in Autophagy and Cell Metabolism

**Figure 6:**
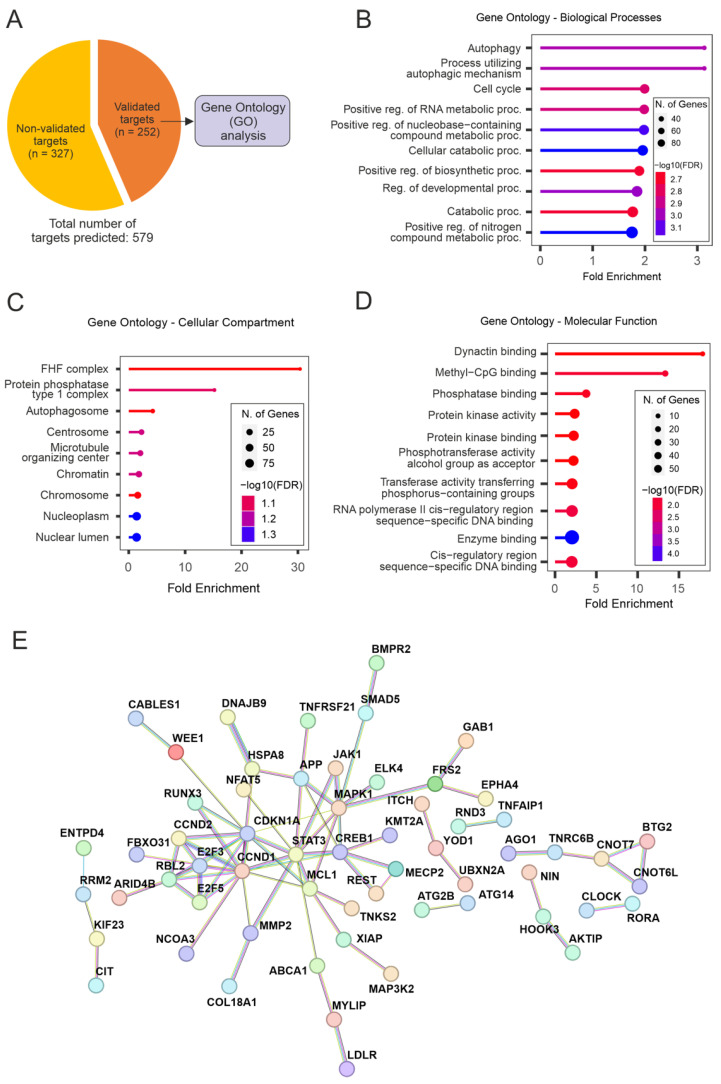
Enrichment analyses for the targets of hsa-miR-17-5p. (**A**) Target prediction results reveal a total of 579 predicted targets for hsa-miR-17-5p—of which 252 have been experimentally validated. The list of validated targets used for all enrichment analyses is presented here. (**B**–**D**) Gene ontology analyses for the targets of hsa-miR-17-5p. These analyses were divided into sub-categories: Biological Processes (**B**), Cellular Component (**C**), and Molecular Function (**D**). (**E**) Protein-protein interaction (PPI) networks for the targets of hsa-miR-17-5p that composed GO-BP enrichment results. STAT3, MAPK1, CREB1, E2F3/5, MCL1, CCND1/2, and CDKN1A appear as important molecular hubs.

Multiple genes within the same pathway can be targeted by miRNAs, resulting in a broader yet specific response. To better understand the molecular processes underlying the pro-regenerative effects observed in this study, we performed target prediction analyses for the mature strand of miR-17 in humans (hsa-miR-17-5p). In total, 579 targets of hsa-miR-5p were computationally predicted based on the miRNA’s seed sequence and the probability of the interactions [16]. Experimentally validated targets were extracted from target prediction results (Figure 6A), aiming to explore the miRNA-target interactions presenting the highest levels of evidence.

Functional annotation analyses (GO) for experimentally validated targets of hsa-miR-17-5p revealed an important enrichment for autophagy- and metabolism-related terms for all ontology analyses performed (GO-Biological Processes [GO-BP], GO-Cellular Component [GO-CC], GO-Molecular Function [GO-MF]. The GO-BP terms “Autophagy” and “Process utilizing autophagic mechanism” were the top results based on Fold Enrichment, followed by “Cell cycle” and a variety of general metabolism-related terms (Figure 6B). The same pattern is observed for GO-CC results, where processes related to cell metabolism/protein metabolism, as well as autophagy, are high in the list of enrichment results (Figure 6C). Remarkably, the term “Microtubule organizing center” figures among the top hits for GO-CC results, indicating that terms enriched for general metabolic pathways are originating in parts from microtubule/cytoskeleton organization processes. This is substantiated by the most enriched term for GO-MF results, “Dynactin binding” (Figure 6D), which is followed by terms related to metabolic pathways and enzymatic activity.

Aiming to further explore the drivers for the functional enrichment presented for the targets of miR-17, protein-protein interaction (PPI) networks were assembled. This was conducted based on the list of targets composing the results for GO-BP analysis (which presented the most comprehensive results in terms of the number of enriched genes per category; Supplementary Material Table S1). These analyses revealed important interacting hubs (Figure 6E), with candidates such as STAT3, MAPK1, CREB1, E2F3/5, MCL1, CCND1/2, and CDKN1A playing a central role in the networks. Overall, functional annotation analyses showed that miR-17 modulates important cellular processes such as autophagy and cell metabolism. An enrichment of cytoskeleton organization processes was also revealed, underlining the evidence for the regenerative effects of miR-17, which was shown in our in vitro studies.

## 3. Discussion

In this study, we used AAV vectors to overexpress miR-17 in primary cortical neurons and investigate its effects on neurite outgrowth and regeneration. We demonstrate that AAV.miR-17 had no effect on neurite outgrowth and arborization. However, in lesion paradigms, AAV.miR-17 enhances neurite and axon regeneration. Furthermore, we show by target prediction and functional annotation analysis that miR-17 is predicted to regulate the mRNAs of genes related to autophagy and cell metabolism. These results extend the knowledge about the intrinsic capacity for axonal regeneration, especially the individual role of miR-17.

The role of miR-17 during CNS development has been evaluated by different studies. Expression levels of miR-17 are high during the early stages of cerebral cortex development and decrease during development [11,17]. In the spinal cord, it has been shown that miR-17-mediated repression of Olig2 mRNA plays a critical role during dorsoventral patterning [18]. During the differentiation of neural progenitor cells, miR-17 favors a neurogenic fate by inhibiting the acquisition of gliogenic competence [11,17]. More recently, it has been shown that miR-17 is involved in the proper positioning of corpus callosum axons within the tract by repression of Epha4 mRNA [19].

Whereas it is known that miR-17 has important roles during CNS development, including in axonal outgrowth, it remains unknown whether miR-17 would also influence axon regeneration. To answer this question, we used AAV vectors to overexpress miR-17 in primary cortical neurons from E18 rats. Although we did not perform any test to confirm the expression of miR-17, the expression of mCherry is a good proxy for the expression of transgenes in the same vector as shown before by us [14]. First, evaluate the effect on neurite outgrowth. We found that forced expression of miR-17 has no influence on neurite outgrowth and arborization.

These results contrast with the study of Zhang et al., which shows that overexpression of the miR-17-92 cluster enhances axonal outgrowth [20]. Although the miR-17 belongs to the miR-17-92 cluster, the cluster also contains five other mature miRNAs, miR-18a, miR-19a, miR-19b, miR-20a, and miR-92a [10], which could also have roles in axonal elongation. Moreover, in the previous study, they used the CAG promoter to drive the expression of the miR-17-92 cluster and a different method of gene transfer (electroporation) [20], which confers a rapid expression of the transgene (~24 h). In our paradigm, we transduced the cortical neurons on DIV0 with AAV vectors and evaluated neurite outgrowth and arborization on DIV8. The expression of transgenes by AAV vectors in primary cortical neurons usually takes 3 days to reach a detectable level, for instance, evaluated by mCherry expression. Thus, in our paradigm, the effect of miR-17 is supposed to start on DIV3 when the neurites have already started to develop, which could explain the lack of effect on neurite outgrowth and arborization and the contrast result compared with the study of Zhang et al. [20].

Interestingly, when we added a lesion paradigm, we found that AAV.miR-17 enhances neurite and axon regeneration. We first performed a scratch lesion on DIV7 and evaluated neurite regeneration 24 h later. In this paradigm, we demonstrated a significant increase in neurite regeneration mediated by AAV.miR-17 compared to the control vector. In a second lesion paradigm, the primary cortical neurons were cultured in a microfluidic device that separates axons from cell bodies and dendrites, allowing selective axonal lesions and evaluation of axonal regeneration [14,15,21]. In this model, in which the lesion was performed on DIV7 to DIV9, we found that AAV.miR-17 enhances axonal regeneration. Importantly, in both injury models, the lesions were conducted at least on DIV7, when the expression of mCherry, which could be used as a proxy for the expression of miR-17, is already at a high level. Therefore, the lack of effect on neurite outgrowth and arborization contrasted with the enhancement of neurite and axonal regeneration mediated by AAV.miR-17 could be explained by the time when the expression of the miR-17 started, which was before the lesion in the injury paradigms. Alternatively, it is known that the expression levels of miR-17 are high during early CNS development and decline with time [11,17]. Consequently, the lack of impact on neurite outgrowth and arborization in our model could also be explained by the fact that the expression levels of miR-17 on the first days of the culture could already be high. On the other hand, as culture develops, the normal expression levels of miR-17 are probably declining, and then, when we overexpressed it by AAV vectors and perform the lesion, we see the effect on neurite and axon regeneration.

In terms of the function of genes targeted by miR-17, we observed that autophagy- and metabolism-related processes are highly enriched in gene ontology analyses. It is well documented that autophagy mechanisms are intrinsically related to axonal regeneration in vitro and in vivo [15,22,23]. Our results showed that GO terms entitled “Autophagy” and “Process utilizing autophagic mechanism” are the most enriched terms for biological processes. Furthermore, it is also remarkable that a few terms directly related to cytoskeleton (re-)organization (“Microtubule organizing center”; “Dynactin binding”) appear high in the gene ontology results for the targets of miR-17, followed by a myriad of processes related to cell metabolism. Microtubule reorganization and actin dynamics are processes known to be heavily dependent on energy metabolism [24] and even to influence the general cell metabolism through tubulin partitioning, for example [25]. Therefore, it is likely that the regenerative effects observed in our AAV.miR-17-transduced neuronal cultures are at least partially related to the overexpression of miR-17, which modulates autophagic and metabolic pathways in injured neurons to promote axonal regeneration.

Finally, interesting hub candidates that appear in PPI network analyses for the targets of miR-17 are known to influence pathways such as autophagy (STAT3, CCND1), cell metabolism (CREB1, MCL1) as well as cytoskeleton organization (MAPK1, STAT3, JAK1) [26,27,28,29,30,31]. In more detail, a substantial number of studies have demonstrated the importance of the aforementioned candidates in autophagy. For instance, STAT3-mediated autophagic mechanisms have been extensively described in recent years. STAT3 inhibition has been shown to promote p62/SQSTM1 degradation LC3 conversion, directly influencing autophagosome formation [32,33,34]. Both CCND1 and CCND2 isoforms have also been linked to autophagy in different disease states and cell types [35,36,37,38]. Targets of miR-17 are also deemed to play a pivotal role in different metabolism-related pathways, hence the important enrichment captured in functional annotation experiments. The CREB family, for example—including CREB1, one of the main hub proteins for the targets of miR-17—are known to be central regulators of cell metabolism. This family of transcription factors directly modulates processes such as lipid metabolism, carbohydrate metabolism, and metabolic inflammation through histone acetylation and transcriptional activation. This has been demonstrated for various tissue types in a number of experimental paradigms [27,39,40,41].

One of the limitations of this study is the lack of validation of the target prediction analyses. While our bioinformatics approach has identified several genes potentially regulated by AAV.miR-17—including several previously validated interactions—we cannot definitively confirm their regulation in our specific experimental model without additional validation approaches. The absence of direct experimental validation means that interpreting the broader implications of our findings must be conducted with caution. Although our data suggest that miR-17 plays a key role in modulating axonal regeneration through autophagic and metabolic pathways, these conclusions should be considered preliminary. Future studies are needed to experimentally verify the predicted targets and elucidate the detailed mechanisms of miR-17 regulation in the context of axonal regeneration in vitro. Despite this limitation, our findings offer valuable evidence for an important involvement of miR-17 in modulating axonal regeneration through autophagic and metabolic pathways and lay the groundwork for future investigations.

Taken together, this study demonstrates that overexpression of miR-17 by AAV vectors enhances neurite and axon regeneration with no influence on neurite outgrowth and arborization. Target prediction and functional annotation analyses associate these effects with the regulation of genes involved in autophagy and cell metabolism. We propose that miR-17-mediated regulation of autophagy is a key mechanism by which it promotes axon regeneration. Therefore, our results suggest that miR-17 is a promising target therapeutic candidate to promote the repair of neurodegenerative disorders, including neurodegenerative diseases and traumatic injuries to the CNS.

## 4. Materials and Methods

### 4.1. Cloning and Production of AAV Vectors

The AAV vector expression plasmid used to overexpress miR-17 (AAV.miR-17) was cloned into the pAAV-hSyn-mCherry plasmid [GenBank ID: KT345943]. The AAV.miR-17 vector expresses the miR-17 precursor (pre-miR-17) under the control of the H1 promoter (Figure 1A). Both the AAV.miR-17 vector and the base vector pAAV-hSyn-mCherry, which served as the control (AAV.CTRL), contain the human synapsin (hSyn) promoter regulating the expression of the mCherry reporter gene. All plasmids were sequenced to verify their correct sequence.

For all experiments, AAV pseudotype 1/2 was used, which consists of an AAV2-derived genome packed into hybrid capsids of AAV1 and a mutated form of the AAV2 capsid [42]. Generation of AAV was performed as reported previously [43]. Briefly, the AAV expression plasmids (pAAV.miR-17 or pAAV.CTRL) and helper plasmids were transfected into HEK-293 cells using calcium phosphate. AAV vectors were purified by virus gradient centrifugation in iodixanol and fast protein liquid chromatography. The virus stocks were tested on primary cortical neurons for transduction efficacy and toxicity, and viral titers were determined using qPCR.

### 4.2. Primary Culture of Cortical Neurons and AAV Vector Transduction

Primary cultures of cortical neurons were prepared from Wistar rats on embryonic day 18 (E18), as described previously [14,15], with the approval of the Animal Use Ethics Committee of the Federal University of Minas Gerais. Briefly, cortices from the embryos were dissected and dissociated with trypsin (Sigma-Aldrich, St. Louis, MO, USA) using a custom Pasteur pipette. After centrifugation, the neurons were resuspended in cortex medium composed of serum-free neurobasal medium supplemented with B-27, L-glutamine, penicillin/streptomycin/neomycin mix (all Thermo-Scientific, Waltham, MA, USA) and transferrin (Sigma-Aldrich) and seeded in 24-well plates pre-coated with poly-L-ornithine and laminin (both Sigma-Aldrich) or inappropriate dishes and incubated at 37 °C and 5% CO_2_. AAV transduction was conducted 4 h after seeding with AAV.CTRL and AAV.miR-17 with 1 × 10^7^ transducing units (TU), resulting in only minor toxicity and equal transduction rates (80–90%) on the day in vitro (DIV) 8, assessed by the expression of mCherry using a fluorescence microscope (Axiovert) equipped with the ZEN software version 1.0 (both Zeiss, Jena, Germany). Medium changes were performed every other day. To evaluate cellular viability after transduction with AAV vectors, neurons (40,000 cells/well) were seeded in 96-well plates. The cells were transduced with either control (AAV.CTRL) or AAV.miR-17 vectors, and on DIV8, the MTT cell viability assay (Thermo-Scientific) was performed.

### 4.3. Neurite Outgrowth Experiment and Quantification

To assess neurite outgrowth in vitro, neurons were seeded on a glass coverslip in 24-well plates (80,000 cells/well) and transduced with either control (AAV.CTRL) or AAV.miR-17. Two coverslips were used per AAV vector per culture. On DIV8, the cells were fixed in 4% paraformaldehyde (PFA, Sigma-Aldrich) in phosphate-buffered saline (PBS), and immunostaining was performed with an antibody against the cytoskeletal protein β-III-tubulin (Tuj-1). Per coverslip, 10 images at 20× magnification were conducted randomly using a fluorescence microscope (Axio Imager Apotome.2) equipped with ZEN software (both Zeiss). Neurite outgrowth was evaluated using ImageJ software (version 2.14.0/1.54f), where the area stained by Tuj1 was measured and normalized by the total number of cells in the image in a blinded fashion.

### 4.4. Evaluation of Neurite Arborization by Sholl Analysis

The complexity of neurite arborization was assessed by Sholl analysis on individualized neurons. Neurons were seeded at a low density (20,000 cells/well) on a glass coverslip in a 24-well plate transduced with AAV.CTRL or AAV.miR-17 vectors, fixed on DIV8 and immunostained with the antibody Tuj-1 as described before. Two coverslips were used per AAV vector per culture. At least 10 isolated neurons per coverslip were imaged using a 40× objective and a fluorescence microscope (Axio Imager Apotome.2) equipped with ZEN software (both Zeiss). The Sholl analysis was conducted using ImageJ software and the plugin Neuroanatomy. The Sholl analysis involves drawing concentric circles around the cell body. The first circle was located 5 µm from the center of the cell body, and the last at a distance of 51 µm, with a 2 µm interval between each circle. The neurite extension intersections with each concentric circle and the total intersections were quantified for each neuron in a blinded fashion, allowing for the measurement of neurite arborization complexity.

### 4.5. Mechanical Scratch Lesion and Quantification of Neurite Regeneration

A mechanical scratch lesion was performed to evaluate neurite regeneration in vitro. Similar to what was described before, neurons were seeded (100,000 cells/well) on a glass coverslip in a 24-well plate and transduced with AAV.CTRL or AAV.miR-17 vectors. On DIV7, a mechanical scratch lesion was made with a pipette tip from one border to the other of the well in the center of the glass coverslip. Twenty-four hours later, the cells were fixed and immunostained with the antibody Tuj-1, as described before. Two coverslips were used per AAV vector per culture. At least 5 images per coverslip were taken in the center of the coverslip using a fluorescence microscope (Axio Imager Apotome.2, Zeiss) at 20× magnification. To trace the regenerating neurites ImageJ software with the NeuronJ plugin was used. Quantifications were made by summing the total length of neurites within 100 to 200 µm from the scratch lesion border. Also, the number of neurites crossing a line at 200 µm from the scratch lesion border was quantified. For both quantifications, the values were normalized by the number of cells located from the scratch lesion border up to 100 µm before the lesion. Due to the high variability of this assay, values for each experiment were normalized to the control group (AAV.CTRL).

### 4.6. Culture of Cortical Neurons in Microfluidic Chambers

Microfluidic chambers [17] were produced and prepared as reported previously [15,16]. The microfluidic chambers used in this study consist of two main channels, called “somatic and axonal compartments”, and microgrooves connecting these two main channels. This setup allows the separation of axons from neuronal cell bodies and dendrites [21]. The microfluidic chambers were mounted on 35 mm glass coverslips (Thermo-Scientific) previously coated with poly-L-lysine (Sigma-Aldrich) at a concentration of 0.1 mg/mL. Primary cortical neurons were prepared as previously described and seeded into the somatic compartment at a density of 4 × 10^5^ cells/chamber. Four hours later, the neurons were transduced using either AAV or AAV.CTRL or AAV.miR-17 vectors. The medium volume on both compartments was changed by half every other day, with a larger medium volume on the somatic compartment to guide axonal growth through the microgrooves.

### 4.7. Axotomy, Live-Imaging, and Quantification of Axonal Regeneration of Primary Cortical Neurons Cultured in Microfluidic Chambers

After DIV7-9, when the axons reached the axonal compartment, an axotomy was performed using a vacuum suction of the culture medium from the axonal compartment. Afterward, the axonal compartment was refilled with fresh cortex medium. Axotomy was confirmed using a transmitted light microscope (Axiovert, Zeiss). For live imaging, regenerating axons were identified by mCherry expression. Regenerating axons were imaged 48 h after axotomy using a 10× objective and a fluorescence microscope (Axiovert, Zeiss) equipped with an incubation system (37 °C and 5% CO_2_). Using the Tile plugin of the ZEN software, the length of the axonal compartment adjacent to 25 microgrooves in the center of the chamber was imaged. The number of regenerating axons was quantified at distances of 100, 250, 500, and 1000 µm from the lesion site in a blinded fashion.

### 4.8. Immunofluorescence

To analyze neurite outgrowth, complexity, and regeneration, immunostaining against β-III-tubulin (Tuj1) was performed. At the end of each experiment, neurons were fixed with 4% PFA in PBS for 10 min. After washing with PBS, the cells were permeabilized with 0.5% Triton X-100 in PBS for 20 min and incubated with blocking solution (5% Normal Goat Serum (Sigma Aldrich) and 1% Bovine Serum Albumin (Sigma Aldrich)) in PBS at room temperature for 1 h. Then, the cells were incubated overnight at 4 °C with the primary antibody Tuj1 (1:10,000; Biolegend (San Diego, CA, USA), #801201), diluted in the same blocking solution. Detection of β-III-tubulin was carried out using the secondary antibody Alexa Fluor 488 (1:1000; Thermo-Scientific) for 1 h at room temperature. Nuclei were counterstained with DAPI (Sigma), and slides were mounted with Fluoromount (Thermo-Scientific) and stored at 4 °C until fluorescence microscopy imaging.

### 4.9. MiRNA-Target Prediction and Gene Ontology Analyses

To investigate the mechanisms by which miR-17 may interfere with axonal regeneration, a bioinformatics analysis of the miR-17 target mRNAs was conducted. Target prediction analysis for the mature strand of human miR-17 (hsa-miR-17-5p) was performed using miRWalk version 3.0 [16]. Experimentally validated targets were extracted using the embedded miRTarBase database [44], a curated source of validated miRNA-target interactions, which are extracted from the literature in the field using natural language processing. A cutoff of 0.8 in the probability score for the miRNA-target interaction was considered for target prediction aiming for miRNA-target interactions with a high level of evidence.

Gene ontology (GO) analysis for validated targets of hsa-miR-17-5p was performed using ShinyGO version 8.0 [45]. Significance for enriched terms was considered at FDR < 0.1, and the pathway redundancy reduction function was used for all enrichment analyses. The top 10 most enriched terms in each GO category (Biological Processes, Cellular Component, Molecular Function) were extracted for display. Protein-protein interaction networks were created with the STRING database v.12.0 [46], using targets enriched for the top 10 most significant pathways in GO-Biological processes results.

### 4.10. Statistical Analysis

Statistical analyses were conducted using GraphPad Prism software (version 9.5.1). All experimental data were pooled into mean ± standard error of the mean. Differences between mean values were analyzed using the following tests: one-sample *t*-test, unpaired *t*-test, and two-way ANOVA, followed by Sidak’s multiple comparisons post-test. Differences were considered significant when the *p*-value was less than 0.05.

## Data Availability

Data is contained within the article or Supplementary Material.

## Supplementary Materials

The following supporting information can be downloaded at: https://www.mdpi.com/article/10.3390/ijms25169057/s1.
